# Panobinostat Synergistically Enhances the Cytotoxic Effects of Cisplatin, Doxorubicin or Etoposide on High-Risk Neuroblastoma Cells

**DOI:** 10.1371/journal.pone.0076662

**Published:** 2013-09-30

**Authors:** Guan Wang, Holly Edwards, J. Timothy Caldwell, Steven A. Buck, William Y. Qing, Jeffrey W. Taub, Yubin Ge, Zhihong Wang

**Affiliations:** 1 Department of Pediatrics, Wayne State University School of Medicine, Detroit, Michigan, United States of America; 2 College of Life Science, Jilin University, Changchun, China; 3 Department of Oncology, Wayne State University School of Medicine, Detroit, Michigan, United States of America; 4 Molecular Therapeutics Program, Barbara Ann Karmanos Cancer Institute, Wayne State University School of Medicine, Detroit, Michigan, United States of America; 5 MD/PhD Program, Wayne State University School of Medicine, Detroit, Michigan, United States of America; 6 Cancer Biology Program, Wayne State University School of Medicine, Detroit, Michigan, United States of America; 7 Division of Pediatric Hematology/Oncology, Children's Hospital of Michigan, Detroit, Michigan, United States of America; Virginia Commonwealth University, United States of America

## Abstract

High-risk neuroblastoma remains a therapeutic challenge with a long-term survival rate of less than 40%. Therefore, new agents are urgently needed to overcome chemotherapy resistance so as to improve the treatment outcome of this deadly disease. Histone deacetylase (HDAC) inhibitors (HDACIs) represent a novel class of anticancer drugs. Recent studies demonstrated that HDACIs can down-regulate the CHK1 pathway by which cancer cells can develop resistance to conventional chemotherapy drugs. This prompted our hypothesis that combining HDACIs with DNA damaging chemotherapeutic drugs for treating neuroblastoma would result in enhanced anti-tumor activities of these drugs. Treatment of high-risk neuroblastoma cell lines with a novel pan-HDACI, panobinostat (LBH589), resulted in dose-dependent growth arrest and apoptosis in 4 high-risk neuroblastoma cell lines. Further, the combination of panobinostat with cisplatin, doxorubicin, or etoposide resulted in highly synergistic antitumor interactions in the high-risk neuroblastoma cell lines, independent of the sequence of drug administration. This was accompanied by cooperative induction of apoptosis. Furthermore, panobinostat treatment resulted in substantial down-regulation of CHK1 and its downstream pathway and abrogation of the G2 cell cycle checkpoint. Synergistic antitumor interactions were also observed when the DNA damaging agents were combined with a CHK1-specific inhibitor, LY2603618. Contrary to panobinostat treatment, LY2603618 treatments neither resulted in abrogation of the G2 cell cycle checkpoint nor enhanced cisplatin, doxorubicin, or etoposide-induced apoptosis in the high-risk neuroblastoma cells. Surprisingly, LY2603618 treatments caused substantial down-regulation of total CDK1. Despite this discrepancy between panobinostat and LY2603618, our results indicate that suppression of the CHK1 pathway by panobinostat is at least partially responsible for the synergistic antitumor interactions between panobinostat and the DNA damaging agents in high-risk neuroblastoma cells. The results of this study provide a rationale for clinical evaluation of the combination of panobinostat and cisplatin, doxorubicin, or etoposide for treating children with high-risk neuroblastoma.

## Introduction

Neuroblastoma is the most common malignant extracranial solid tumor of childhood and accounts for approximately 11% of all pediatric cancers and 15% of all pediatric cancer deaths [1], [2]. Approximately 650 new cases are diagnosed in the United States annually with a peak incidence in early childhood (ages 0–4 years). Neuroblastoma remains a major therapeutic challenge despite decades of intensive research and therapeutic trials. With current treatment protocols, including high-dose chemotherapy with autologous stem cell transplantation/peripheral stem cell transplantation, radiation, and surgery, patients with high-risk metastatic neuroblastoma have long-term survival rates of less than 40% [1], [2]. This highlights the chemotherapy-resistant nature of this tumor. Therefore, new agents are urgently needed to overcome chemotherapy resistance so as to improve the treatment outcome of this deadly disease in children.

The frontline chemotherapeutic drugs (e.g., etoposide, doxorubicin and cisplatin) for treating neuroblastoma are all DNA damaging agents, which induce DNA damage to exert their anti-tumor activities [3]–[5]. These DNA lesions elicit activation of cell cycle checkpoints, controlled by the ATM and ATR kinases [6], and CHK1 and CHK2 are key downstream checkpoint substrates of ATM and ATR [7]. This allows repair of DNA damage before it is replicated and passed on to daughter cells. Therefore, abrogation of the DNA damage checkpoints would limit the time of repair of DNA lesions, thus promoting apoptosis [8]. CHK1 contributes to all currently defined cell cycle checkpoints [8]. It has been documented that inhibition of CHK1 with pharmacologic intervention or by siRNA knockdown sensitizes cancer cells including neuroblastoma cells to S/G2-phase-acting agents [8], [9].

Histone deacetylase (HDAC) inhibitors (HDACIs) are a promising new class of anti-cancer drugs. HDACI induce cell cycle arrest, differentiation and apoptosis in cancer cells, but less so in normal cells [10]. Despite their well-characterized molecular and cellular effects, single-agent clinical activities of HDACIs have been modest [11]–[18]. Thus, there would seem to be compelling rationale for developing rationally designed drug combinations using HDACIs in combination with other chemotherapy agents. A recent study showed that HDACIs down-regulate expression of CHK1 in non-small cell lung cancer cells [19]. These results prompted our hypothesis that HDACIs may suppress the CHK1 pathway in high-risk neuroblastoma cells to enhance the cytoxicities of etoposide, doxorubicin, or cisplatin.

In this project, we demonstrated that the novel pan-HDACI, panobinostat [14], substantially repressed the expression of CHK1 leading to abrogation of the G2 cell cycle checkpoint and synergistically enhanced the cytotoxic effects of etoposide, doxorubicin, or cisplatin, on high-risk neuroblastoma cells. Although there is a need of follow up studies in *in vivo* models, our results suggest that integrating panobinostat into the conventional chemotherapy of high-risk neuroblastoma may improve treatment efficacy.

## Materials and Methods

### Drugs

Etoposide, doxorubicin, and cisplatin were purchased from Sigma-Aldrich (St Louis, MO). LY2603618 and panobinostat were purchased from Selleck Chemicals (Houston, TX).

### Cell Culture

The SK-N-AS, SK-N-DZ, SK-N-SH and SK-N-BE(2) human cell lines derived from patients with high-risk neuroblastoma were purchased from the American Type Culture Collection (ATCC; Manassas, VA). The SK-N-AS and SK-N-DZ cell lines were cultured in Dulbecco's Modified Eagle Medium (DMEM, Invitrogen, Carlsbad, CA), while the SK-N-SH and SK-N-BE(2) cell lines were cultured in RPMI-1640 (Invitrogen) with 10% heat-inactivated fetal bovine serum (FBS; Hyclone Labs, Logan, UT) plus 100 U/mL penicillin and 100 µg/mL streptomycin in a 37°C humidified atmosphere containing 5% CO2/95% air.

### *In Vitro* Cytotoxicity Assays

*In vitro* drug cytotoxicities of neuroblastoma cell lines were measured by using MTT (3-[4,5-dimethyl-thiazol-2-yl]-2,5-diphenyltetrazolium-bromide, Sigma-Aldrich) reagent, as previously described [20]–[22]. Briefly, SK-N-AS, SK-N-DZ, SK-N-SH or SK-N-BE(2) cells were cultured in 100 µl of DMEM/RPMI-1640 with 10% FBS in 96-well plates. Cells were incubated at 37°C in the presence of variable concentrations of etoposide (0–32 µM), doxorubicin (0–4 µM), or cisplatin (0–32 µM) with or without panobinostat (2.5–20 nM). After 44 hours, MTT was added to a final concentration of 1 mM. After 4 hours, formazan crystals were dissolved by the addition of 100 µl of 10% SDS with 10 mM HCl. Optical densities were measured with a visible microplate reader at 590 nm. IC_50_ values were calculated as drug concentrations necessary to inhibit 50% growth compared to untreated control cells. The results are presented as means ± standard errors from at least 3 independent experiments. The extent and direction of antitumor interactions between panobinostat and etoposide, doxocubin or cisplatin were evaluated by standard isobologram analysis as described previously [20], [21], and by using the CompuSyn software (ComboSyn, Inc., Paramus, NJ). Briefly, drug interactions were quantified by determining the combination index (CI), where CI<1, CI = 1, and CI>1 indicate synergistic, additive, and antagonistic effects, respectively.

### Assessment of Baseline and Drug-Induced Apoptosis

The SK-N-AS, SK-N-DZ, SK-N-SH or SK-N-BE(2) cells treated with variable concentrations of panobinostat (2.5–80 nM) for 48 hours, the SK-N-SH cells treated with etoposide (0.5 µM), doxorubicin (0.05 µM) or cisplatin (1 µM) in the presence or absence of panobinostat (20 nM) for 48 hours, or the SK-N-BE(2) cells treated with etoposide (1 µM), doxorubicin (0.2 µM) or cisplatin (1 µM) in the presence or absence of panobinostat (20 nM) for 48 hours were harvested and thoroughly pipetted. Samples were then taken to determine baseline and drug-induced apoptosis by Annexin V–FITC/Propidium Iodide (PI) (Beckman Coulter; Brea, CA) double staining or PI staining and flow cytometry analysis using a FACS Calibur flow cytometer (Becton Dickinson, San Jose, CA), as described previously [20]. Apoptotic events were expressed as the percent of Annexin V+ cells or the percent of subG1 cells.

### Effects of Drug Treatments on Cell Cycle Progression

The SK-N-AS, SK-N-DZ, SK-N-SH or SK-N-BE(2) cells treated with panobinostat (2.5–80 nM) for 48 hours, the SK-N-SH cells treated with etoposide (0.5 µM), doxorubicin (0.05 µM), or cisplatin (1 µM) in the presence or absence of panobinostat (20 nM) for 48 hours, or the SK-N-BE(2) cells treated with etoposide (1 µM), doxorubicin (0.2 µM) or cisplatin (1 µM) in the presence or absence of panobinostat (20 nM) for 48 hours were harvested and fixed with ice-cold 70% (v/v) ethanol for 24 hours. After centrifugation at 200×g for 5 minutes, the cell pellets were washed with PBS (pH 7.4) and resuspended in PBS containing PI (50 µg/mL), Triton X-100 (0.1%, v/v), and DNase-free RNase (1 µg/mL). DNA contents were determined by flow cytometry (FACS Calibur). Cell cycle analysis was performed with the ModFit LTTM3.0 DNA analysis software (Becton Dickinson).

### Western Blot Analysis

Soluble proteins were extracted from the SK-N-AS, SK-N-DZ, SK-N-SH or SK-N-BE(2) cells untreated, or treated with vehicle control or drugs for 48 hours and subjected to SDS-polyacrylamide gel electrophoresis. Separated proteins were electrophoretically transferred onto polyvinylidene difluoride (PVDF) membranes (Thermo Fisher Inc., Rockford, IL) and immunoblotted with anti-α-tubulin, -caspase3, -PARP, -pCDC25C(S216), -pCDK1(Y15), -CDK1, -cyclin B1, (Cell Signaling Technology, Beverly, MA), -CHK1 (Santa Cruz Biotechnology, Santa Cruz, CA), -ac-histone H4, -histone H4 (Upstate Biotechnology, Lake Placid, NY), -acetyl-α-tubulin or -beta-actin antibody (Sigma-Aldrich), as described previously [20], [21]. Immunoreactive proteins were visualized using the Odyssey Infrared Imaging System (Li-Cor, Lincoln, NE), as described by the manufacturer.

### Statistical Analysis

Differences in cell death/apoptosis between etoposide, doxorubicin, cisplatin or panobinostat treated (individually or combined) and untreated cells were compared using the pair-wise two-sample t-test. Statistical analyses were performed with GraphPad Prism 5.0.

## Results

### HDAC Expression and Panobinostat Cytotoxicities in High-Risk Neuroblastoma Cell Lines

**Figure 1:**
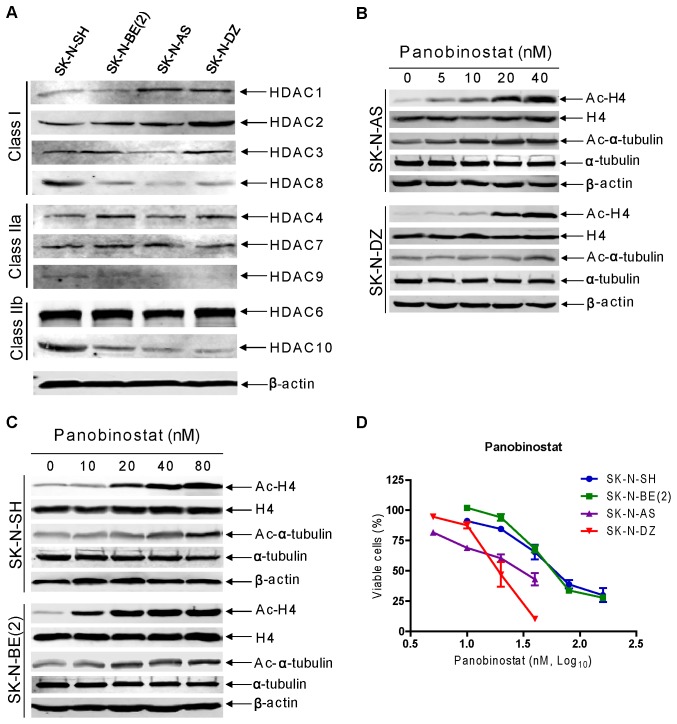
HDAC expression and the effect of panobinostat on high-risk neuroblastoma cell lines. **Panel A:** Protein extracts from log phase SK-N-AS, SK-N-DZ, SK-N-SH and SK-N-BE(2) cells were subjected to Western blots probed by anti-HDAC or -β-actin antibody. **Panels B and C:** SK-N-AS, SK-N-DZ, SK-N-SH or SK-N-BE(2) cells treated with a range of concentrations of panobinostat (0–80 nM) for 48 h were harvested and lysed. Soluble proteins were analyzed on Western blots probed by anti-acetylated (ac)-H4, -H4, -ac-tubulin, -α-tubulin or –β-actin antibody. **Panel D:** SK-N-AS, SK-N-DZ, SK-N-SH or SK-N-BE(2) cells were cultured with a range of concentrations of panobinostat in complete medium in 96-well plates at 37°C for 48 h, cell viabilities were determined using the MTT reagent and a visible light microplate reader. The IC_50_ values were calculated as the concentrations of drug necessary to inhibit 50% growth compared to control cells treated with vehicle control. The data are presented as mean values ± standard errors from at least 3 independent experiments.

Expression of classes I and II HDACs was determined by Western blotting in the SK-N-AS, SK-N-DZ, SK-N-SH and SK-N-BE(2) cell lines, derived from patients with high-risk neuroblastoma. The majority of classes I and II HDACs (except for HDAC5, not shown) were detected in these cell lines, though the levels were variable (Figure 1A). The class III HDACs (SIRTs 1-7) are not targeted by traditional HDACIs [23] and were not included in this experiment. Panobinostat treatment for 48 hours resulted in dose-dependent hyperacetylation of histone H4 and alpha-tubulin (a HDAC6 substrate), however to a lesser extent. In contrast, panobinostat treatment had no effect on total H4 protein levels in all the cell lines (Figures 1B&C). Furthermore, treatment with panobinostat for 48 hours resulted in inhibition of cell proliferation with an IC_50_ of 27.4 nM, 21.9 nM, 72.3 nM, 75.4 nM in the SK-N-AS, SK-N-DZ, SK-N-SH and SK-N-BE(2) cell lines, respectively (Figure 1D). These results demonstrate that the high-risk neuroblastoma cells are sensitive to panobinostat *in vitro*. It is important to note that sensitivities to panobinostat for these high-risk neuroblastoma cell lines are independent of the status of MYCN amplification [24]–[27].

### Induction of Apoptosis by Panobinostat in High-risk Neuroblastoma Cell Lines

**Figure 2:**
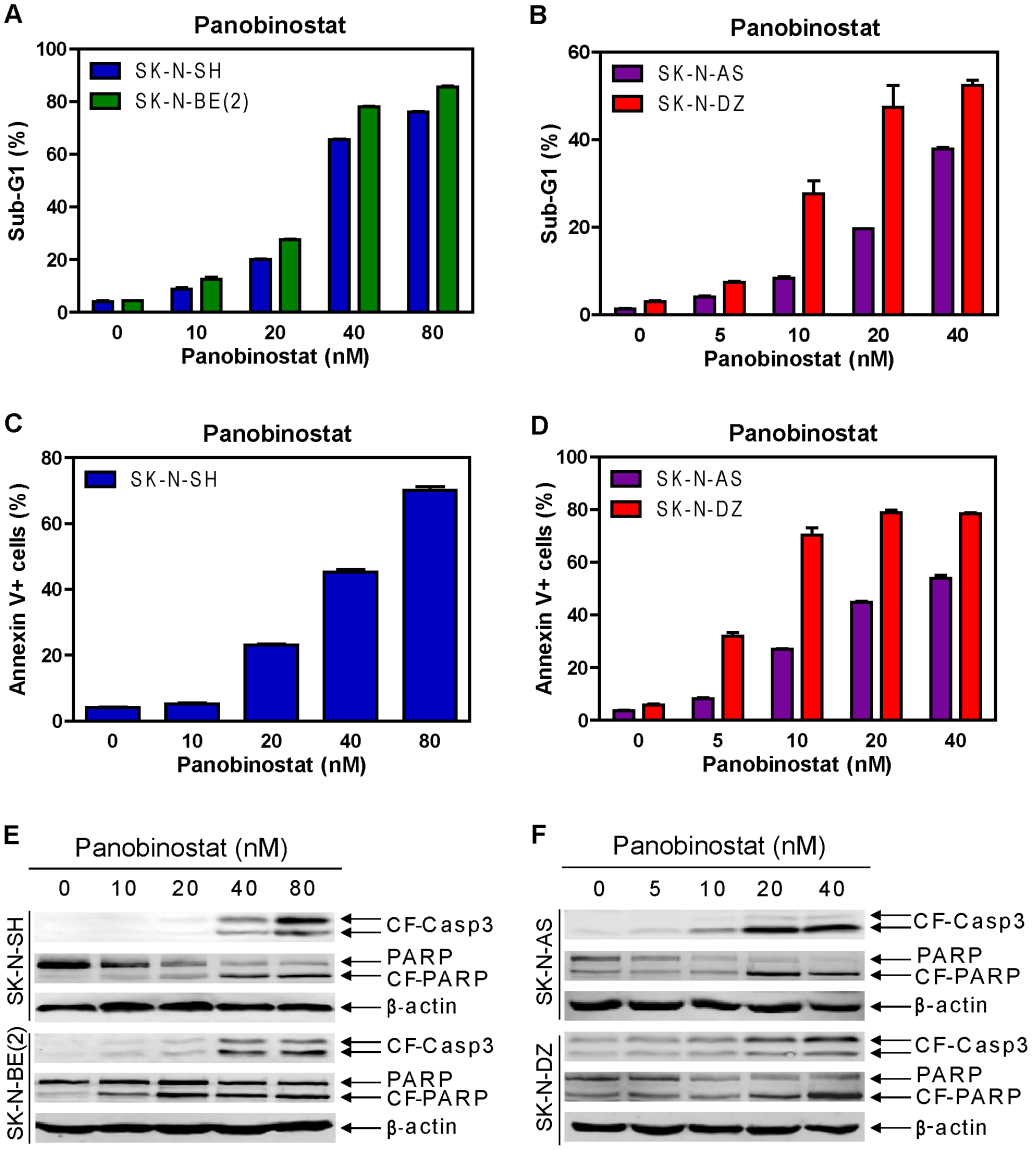
Panobinostat treatments potently induced apoptosis in high-risk neuroblastoma cell lines. **Panels A and B:** SK-N-AS, SK-N-DZ, SK-N-SH or SK-N-BE(2) cell lines were treated with variable concentrations of panobinostat for 48 h. Apoptosis was measured by detecting sub-G1 population with propidium iodide (PI) staining and flow cytometry analyses. The experiment was repeated twice and the results are presented as means ± standard errors of triplicates from one representative experiment. **Panels C and D:** SK-N-AS, SK-N-DZ or SK-N-SH cells were treated with variable concentrations of panobinostat for 48 h. Apoptosis was measured by Annexin V-FITC and PI double staining and flow cytometry analyses. The results are presented as means ± standard errors of triplicates from one representative experiment. **Panels E and F:** SK-N-AS, SK-N-DZ, SK-N-SH or SK-N-BE(2) cells treated with variable concentrations of panobinostat for 48 h were harvested and lysed. Soluble proteins were analyzed on Western blots probed by anti-cleaved form caspase3 (-CF-Casp3), -PARP, or –β-actin antibody.

To determine if the cytotoxic effects of panobinostat were due to induction of apoptosis, the high-risk neuroblastoma cell lines were treated with variable concentrations of panobinostat for 48 hours and then apoptosis was determined by PI staining and flow cytometry analysis. As shown in Figures 2A&B, panobinostat potently induced apoptosis in a dose-dependent fashion in all the cell lines. Representative histograms for the SK-N-DZ line can be found in Figure S1. This was accompanied by dose-dependent cleavage of caspase 3 and PARP (Figures 2E&F). Essentially the same results were obtained in the SK-N-AS, SK-N-DZ, and SK-N-SH cells double stained with Annexin V-FITC and PI (Figures 2C&D). Surprisingly, the SK-N-BE(2) cells post panobinostat treatment could not be stained with Annexin V-FITC due to unknown reasons. Based on these results, we decided to use PI staining and flow cytometry analysis of the sub-G1 population to measure apoptosis for the rest of the study. In addition, panobinostat treatment also increased the proportion of S and G2/M populations in the remaining cells (Table 1). These results demonstrate that panobinostat induces apoptosis and cell cycle arrest to exert its cytotoxic effects on high-risk neuroblastoma cells.

**Table 1:** Cell cycle distribution of non-sub-G1 cells after panobinostat treatment of neuroblastoma cell lines.

Cell	Cell	Panobinostat (nM)					
line	Cycle	0	5	10	20	40	80
	G0/G1	71.76±0.31	ND	68.79±0.64	67.36±0.41	55.02±0.81	51.46±0.48
**SK-N-SH**	**S**	17.04±0.20	ND	16.08±0.15	16.65±0.22	27.33±0.91	29.69±0.62
	**G2/M**	11.61±0.07	ND	15.27±0.36	16.02±0.41	16.91±0.77	18.59±0.41
	**G0/G1**	67.10±0.49	ND	62.77±0.28	55.00±1.36	29.72±0.51	43.76±0.28
**SK-N-BE(2)**	**S**	17.58±0.21	ND	19.08±0.74	23.81±0.69	30.72±0.22	29.00±0.76
	**G2/M**	15.51±0.28	ND	18.30±0.53	22.00±0.37	39.61±0.28	27.62±0.69
	**G0/G1**	60.95 ±0.35	73.55 ±0.23	57.05 ±0.32	42.99 ±0.21	37.65 ±0.25	ND
**SK-N-AS**	**S**	22.14 ±0.38	13.50 ±0.19	16.02 ±0.19	16.84 ±0.57	12.87 ±0.26	ND
	**G2/M**	17.07 ±0.13	13.32 ±0.11	27.60 ±0.37	40.78 ±0.44	50.19 ±0.37	ND
	**G0/G1**	54.81±0.38	42.05±0.58	31.60±0.38	31.39±1.82	32.25±0.83	ND
**SK-N-DZ**	**S**	30.40±0.32	35.35±0.20	34.31±1.28	29.63±0.89	27.00±0.71	ND
	**G2/M**	14.75±0.03	22.34±0.52	31.56±1.30	35.62±1.08	38.68±1.36	ND

**Note:** SK-N-AS, SK-N-DZ, SK-N-SH or SK-N-BE(2) cells were treated with variable concentrations of panobinostat for 48 h. Cell cycle progression was determined by PI staining and flow cytometry analyses. The data are presented as means ± standard errors of triplicates from one representative experiment. ND, not determined.

### Synergistic Anti-tumor Interactions between Panobinostat and Etoposide, Doxorubicin, or Cisplatin in High-risk Neuroblastoma Cell Lines

**Figure 3:**
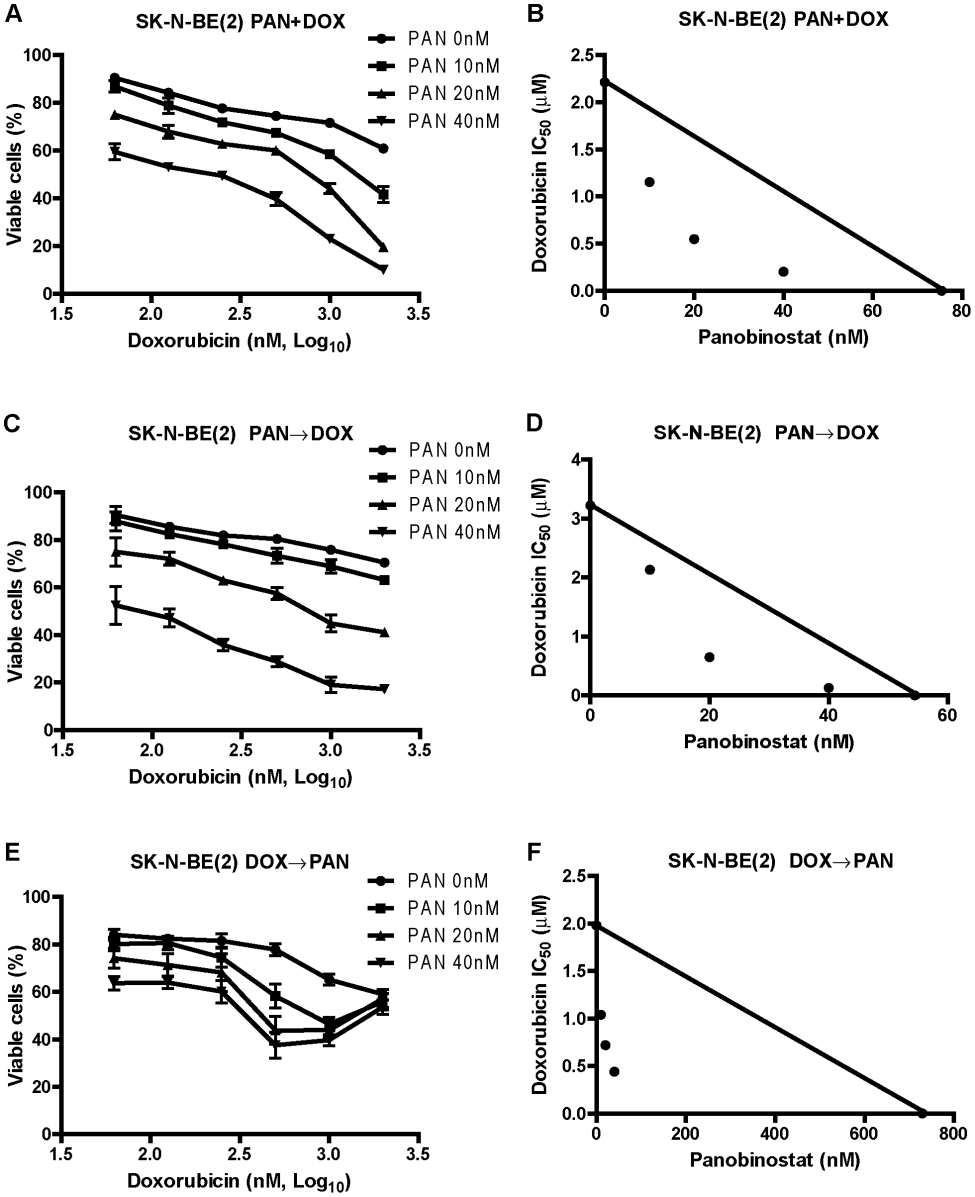
Synergistic antitumor interactions between panobinostat and doxorubicin in SK-N-BE(2) cells. **Panels A, C, and E:** The SK-N-BE(2) cells were treated with variable concentrations of panobinostat and doxorubicin with three different administration schedules including simultaneous treatment with the two drugs for 48 h (designated PAN+DOX, panel A), pretreatment with panobinostat for 24 h followed by simultaneous treatment with the two drugs for 24 h (designated PAN→DOX, panel C), and pretreatment with doxorubicin for 24 h followed simultaneous treatment with the two drugs for 24 h (designated DOX→PAN, panel E). Viable cells were measured by MTT assays and the data are presented as means ± standard errors from at least three independent experiments. **Panels B, D, and F:** Standard isobologram analyses of antitumor interactions between panobinostat and doxorubicin were performed in the SK-N-BE(2) cells treated under the three different administration schedules described above. The IC_50_ values of each drug are plotted on the axes; the solid line represents additive effect, while the points represent the concentrations of each drug resulting in 50% inhibition of growth. Points falling below the line indicate synergism, whereas those above the line indicate antagonism. PAN, panobinostat; DOX, doxorubicin. The same abbreviations were used throughout the study unless otherwise stated.

Next we used MTT assays, standard isobologram analysis, and the CompuSyn software to determine the extent and direction of antitumor interactions between panobinostat and doxorubicin in the SK-N-BE(2) cells. Three different drug administration schedules were used including panobinostat pretreatment for 24 hours followed by simultaneous treatment with the two drugs for 24 hours, doxorubicin pretreatment for 24 hours followed by simultaneous treatment with the two drugs for 24 hours, and simultaneous treatment with the two drugs for 48 hours. Interestingly, at lower doxorubicin doses, panobinostat potently and synergistically enhanced the cytoxicities of doxorubicin independent of the drug administration schedule in the SK-N-BE(2) cell line (Figure 3 and Table 2). In the doxorubicin-first treatment, higher doses of doxorubicin unexpectedly decreased any additional effect of panobinostat. We chose simultaneous drug administration schedule for further experiments. Analogous results were obtained in the SK-N-BE(2) cells simultaneously treated with panobinostat and cisplatin or etoposide for 48 hours (Table 2). When administered simultaneously, synergistic interactions between panobinostat and cisplatin, doxorubicin, or etoposide were also observed in the SK-N-AS, SK-N-DZ, and SK-N-SH cell lines (Table 2). These results demonstrate that panobinostat can synergistically enhance the cytotoxicities of standard DNA damaging chemotherapeutic drugs in high-risk neuroblastoma cells.

**Table 2:** Effects of panobinostat on the cytotoxicities of etoposide, doxorubicin, or cisplatin against high-risk neuroblastoma cell lines.

Cell	DNA damaging	IC_50_ of DNA damaging agents (µM) in the absence or presence of panobinostat (nM)					*P*
line	agents	0	5	10	20	40	*Value*
	Etoposide	2.20±0.20	ND	1.31±0.22 (0.74)	1.00±0.22 (0.74)	0.60±0.12 (0.84)	<0.05
**SK-N-SH**	**Doxorubicin**	0.43±0.03	ND	0.27±0.04 (0.77)	0.16±0.01 (0.65)	0.08±0.01 (0.76)	<0.05
	**Cisplatin**	3.08±0.14	ND	1.56±0.14 (0.65)	1.11±0.19 (0.66)	0.60±0.27 (0.78)	<0.005
	**Etoposide**	21.53±2.42	ND	13.89±0.44 (0.78)	8.63±0.30 (0.67)	3.00±0.35 (0.67)	<0.05
**SK-N-BE(2)**	**Doxorubicin**	2.21±0.03	ND	1.15±0.13 (0.65)	0.55±0.03 (0.51)	0.20±0.02 (0.62)	<0.005
	**Cisplatin**	26.39±1.28	ND	14.67±1.43 (0.69)	7.02±1.08 (0.53)	1.84±0.30 (0.60)	<0.005
	**Etoposide**	36.16±2.89	14.02±0.68 (0.57)	9.23±2.13 (0.62)	7.29±1.88 (0.93)	ND	<0.005
**SK-N-AS**	**Doxorubicin**	0.89±0.08	0.45±0.01 (0.69)	0.30±0.01 (0.70)	0.25±0.01 (1.01)	ND	<0.05
	**Cisplatin**	12.20±0.68	7.89±0.60 (0.83)	5.63±0.49 (0.83)	3.10±0.87 (0.98)	ND	<0.05
	**Etoposide**	27.43±3.33	8.50±1.54 (0.54)	5.27±0.78 (0.65)	1.87±0.53 (0.99)	ND	<0.005
**SK-N-DZ**	**Doxorubicin**	1.38±0.15	0.91±0.11 (0.89)	0.54±0.08 (0.85)	0.15±0.03 (1.03)	ND	<0.05
	**Cisplatin**	26.09±1.67	9.40±1.90 (0.59)	4.95± 1.53 (0.65)	0.87±0.39 (0.95)	ND	<0.005

**Note:** SK-N-AS, SK-N-DZ, SK-N-SH or SK-N-BE(2) cells were treated with variable concentrations of etoposide, doxorubicin or cisplatin for 48 h in the absence or presence of variable concentrations of panobinostat administered simultaneously. Viable cells were measured by MTT assays and the extent and direction of antitumor interactions between panobinostat and etoposide, doxorubicin, or cisplatin were determined by using the CompuSyn software. The numbers in the brackets represent for the combination index (CI) values, where CI<1, CI = 1, and CI>1 indicate synergistic, additive, and antagonistic effects, respectively. The data are presented as means±standard errors from at least 3 independent experiments. ND, not determined.

### Cooperative Induction of Apoptosis by Panobinostat and Etoposide, Doxorubicin, or Cisplatin in High-risk Neuroblastoma Cell Lines

**Figure 4:**
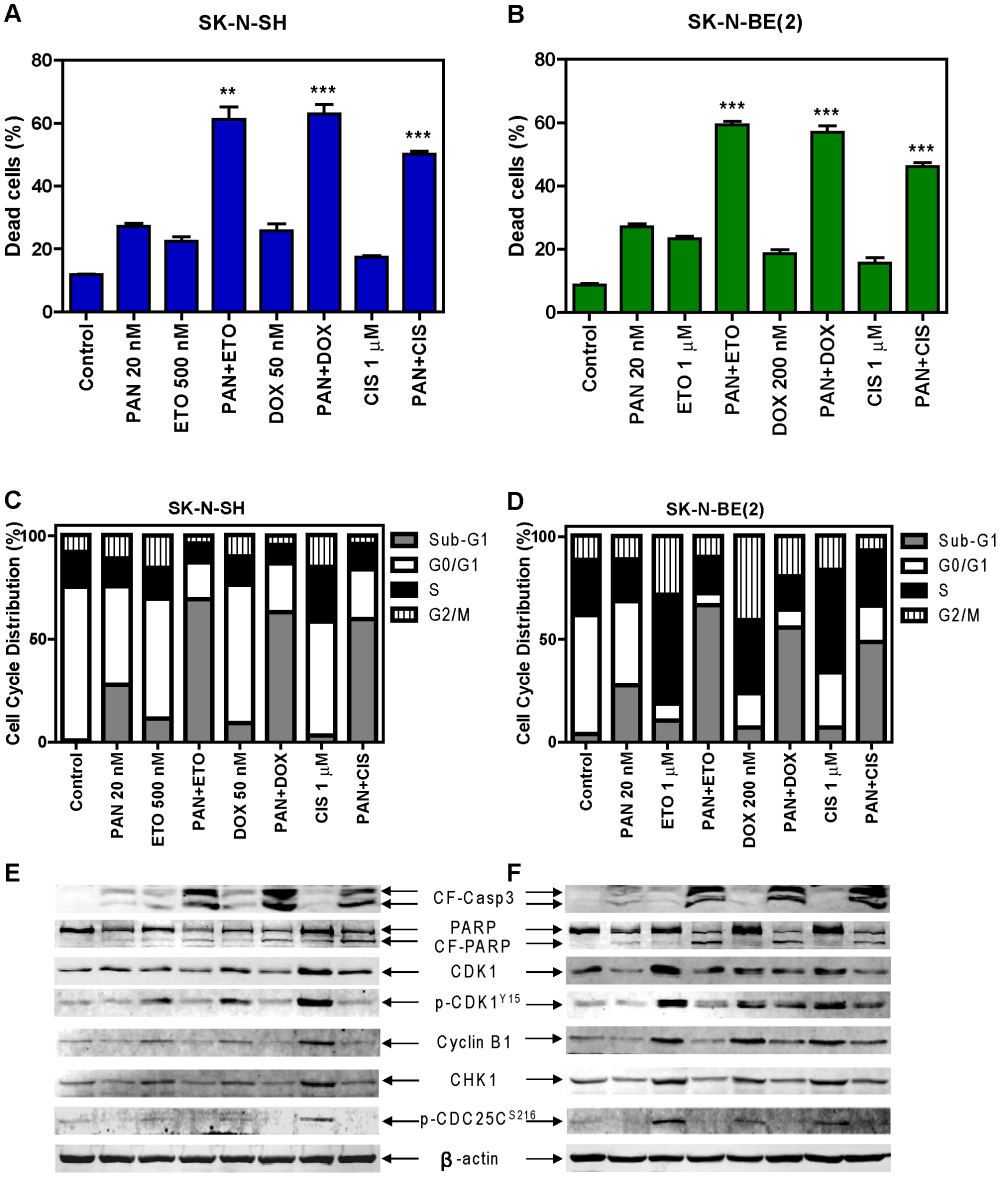
Panobinostat potently enhanced apoptosis induced by etoposide, doxorubicin or cisplatin in SK-N-SH and SK-N-BE(2) cells. SK-N-SH and SK-N-BE(2) cells were treated with etoposide, doxorubicin or cisplatin for 48 h in the absence or presence of 20 nM panobinostat administered simultaneously. Cell death was determined by trypan blue exclusion (**panels A and B**), while apoptosis was measured by PI staining and flow cytometry analyses and Western blotting measuring cleavage of caspase3 and PARP (**panels C-F**). Cell cycle progression was determined by PI staining and flow cytometry analyses (**panels C and D**). Changes in the CHK1-CDK1 signaling pathway were measured by probing Western blots with phosphorylation-specific antibodies. Results from trypan blue exclusion are presented as mean percentages ± standard errors (relative to control cells treated with vehicle control) from three independent experiments. ** indicates p<0.005, while *** indicates p<0.0005. The PI staining and flow cytometry analysis experiment was repeated two times and the data are presented as means of triplicates from one representative experiment. ETO, etoposide; CIS, cisplatin. The same abbreviations were used throughout the study unless otherwise stated.

To determine if the combinations of panobinostat and etoposide, doxorubicin or cisplatin induce cell death/apoptosis in the high-risk neuroblastoma cells, SK-N-BE(2) and SK-N-SH cell lines were treated with etoposide, doxorubicin or cisplatin in the absence or presence of panobinostat for 48 hours. The doses of the drugs used in this experiment were based on the IC_50_ values and the maximum plasma concentrations of each drug [28]-[30]. Cell death was determined by trypan blue exclusion, while apoptosis was determined by PI staining and flow cytometry analysis. Consistent with the results shown in Figure 3 and Table 2, panobinostat potently and significantly enhanced cell death induced by the standard chemotherapeutic drugs (Figures 4A&B). PI staining and flow cytometry analyses demonstrated that apoptosis was the main mechanism of cell death induced by these drug treatments (Figures 4C&D). This was accompanied by substantially increased cleavage of caspase 3 and PARP in the combined drug treatments compared to individual drug treatments (Figures 4E&F). Annexin V-FITC and PI double staining and flow cytometry analysis were also performed with the SK-N-SH cell line and similar results were obtained (data not shown). These results provide compelling evidence that panobinostat potently enhances apoptosis induced by the standard chemotherapeutic drugs in high-risk neuroblastoma cells.

### Abrogation of the S and/or G2 Cell Cycle Checkpoints by Panobinostat in High-risk Neuroblastoma Cell Lines

To begin to determine the molecular mechanisms underlying the synergistic antitumor activities of panobinostat and the standard chemotherapeutic drugs, cell cycle progression in the SK-N-BE(2) and SK-N-SH cells post drug treatments was determined. In the SK-N-SH cells, treatments with the standard chemotherapeutic drugs mainly caused G2/M cell cycle arrest, which was abrogated by the simultaneous administration of panobinostat (Figure 4C). In the SK-N-BE(2) cells, treatments with the standard chemotherapeutic drugs mainly caused S and G2/M cell cycle arrest, which were also abrogated by the addition of panobinostat (Figure 4D). Consistent with the induction of S and/or G2/M arrest, treatments of the cell lines with the standard chemotherapeutic drugs caused increased phosphorylation of CDK1(Y15)[p-CDK1(Y15)], which was at least partially abolished by the addition of panobinostat (Figures 4E&F). This was accompanied by decreased expression of CHK1 and its downstream signaling, e.g., phosphorylation of CDC25C(S216)[p-CDC25C(S216)]. Treatment with panobinostat in SK-N-BE(2) cells resulted in a decrease of total CDK1 levels. These results suggest that decreased activity of the CHK1 pathway may be important for the synergistic anti-tumor activities of the combination of panobinostat and etoposide, doxorubicin or cisplatin in high-risk neuroblastoma cell lines.

### Effects of CHK1 Inhibition on the Cytotoxicities of Etoposide, Doxorubicin, or Cisplatin in High-risk Neuroblastoma Cell Lines

**Figure 5:**
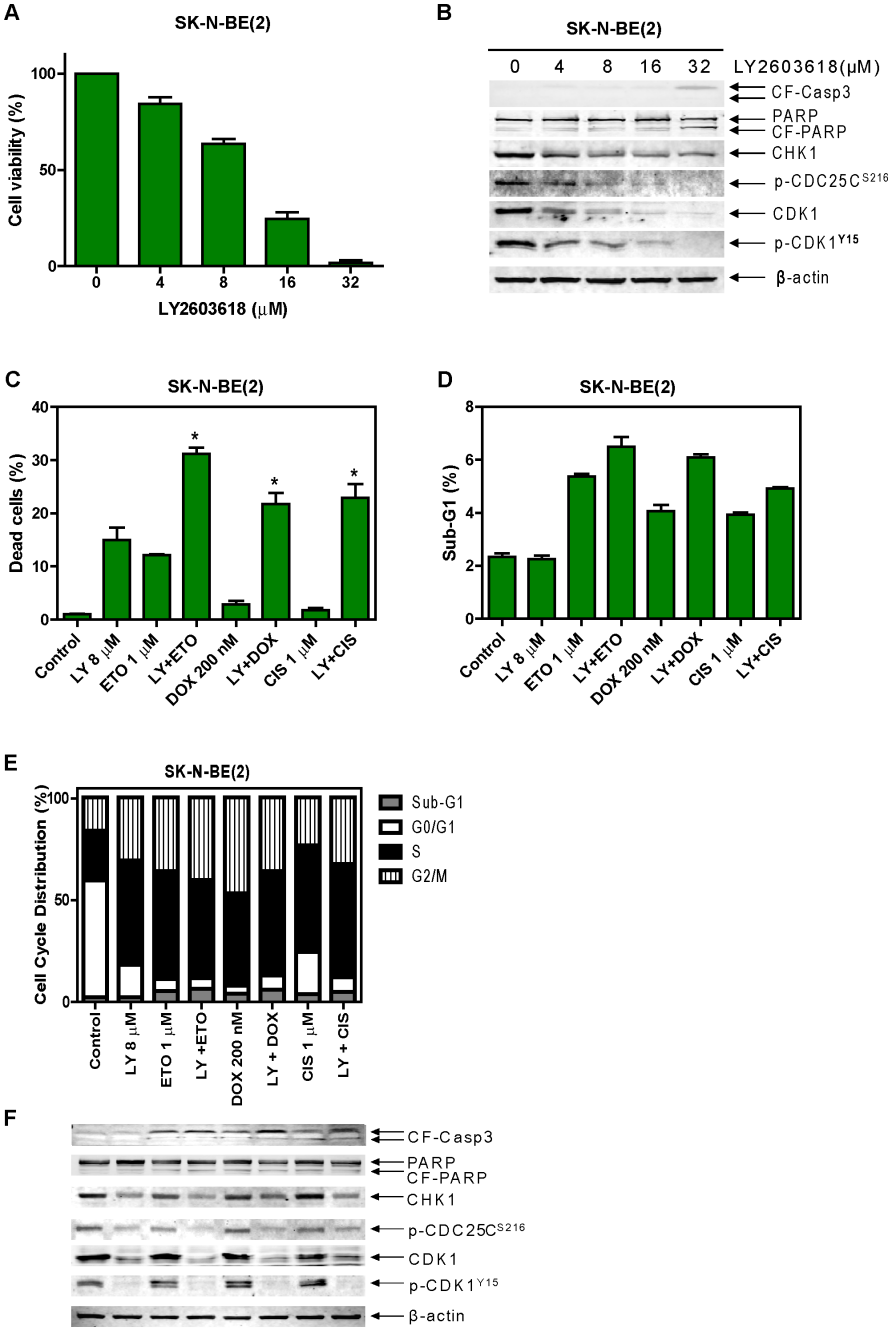
Effects of LY2603618 on the cytotoxicities of cisplatin, doxorubicin, or etoposide in SK-N-BE(2) cells. **Panel A:** SK-N-BE(2) cells were treated with variable concentrations of LY2603618 for 48 h and cell viabilities were determined by MTT assays. The data are presented as means ± standard errors from three independent experiments. **Panel B:** SK-N-BE(2) cells treated with variable concentrations of LY2603618 for 48 h were harvested and subjected to Western blots probed by anti-CF-Casp3, -PARP, -CHK1, -p-CDC25C^S216^, -CDK1, -p-CDK1^Y15^, or –β-actin antibody. **Panels C-F:** The SK-N-BE(2) cells were treated with etoposide, doxorubicin, or cisplatin for 48 h in the absence or presence of 8 µM LY2603618 administered simultaneously. Cell death was determined by trypan blue exclusion (**panel C**), while apoptosis and cell cycle progression were determined by PI staining and flow cytometry analyses (**panels D and E**). Soluble proteins were subjected to Western blots probed by anti-CF-Casp3, -PARP, -CHK1, -p-CDC25C^S216^, -CDK1, -p-CDK1^Y15^, or -β-actin antibody (**panel F**). The trypan blue exclusion data are presented as means ± standard errors from three independent experiments, while the PI staining and flow cytometry analysis experiment was repeated two times and the data are presented as means of triplicates from one representative experiment. * indicate p<0.05. LY, LY2603618.

To further determine whether activity of the CHK1 pathway plays a role in the synergistic antitumor interactions between panobinostat and the standard chemotherapeutic drugs in high-risk neuroblastoma cells, we used a CHK1-specific inhibitor, LY2603618 [31]. Treatments of the SK-N-BE(2) cells with variable concentrations of LY2603618 resulted in dose-dependent inhibition of cell growth determined by MTT assays with an IC_50_ of 10.81 µM (Figure 5A and Table 3). In contrast, LY2603618 only induced very limited amount of apoptosis even at the highest dose used in this experiment (32 µM), reflected by the limited cleavage of caspase 3 and PARP (Figure 5B). As expected, LY2603618 treatments of the SK-N-BE(2) cells resulted in dose-dependent decrease of p-CDC25C(S216) and p-CDK1(Y15) (Figure 5B). Surprisingly, LY2603618 treatments also caused dose-dependent decrease of total CDK1.

**Table 3:** Effects of LY2603618 on the cytotoxicities of etoposide, doxorubicin, or cisplatin against high-risk neuroblastoma cell lines.

Cell	LY260318	DNA damaging	IC_50_ of DNA damaging agents (µM) in the absence or presence of LY2603618 (µM)			*p*
line	IC_50_ (µM)	agents	0	4	8	value
		**Etoposide**	1.85±0.09	0.57±0.01 (0.64)	0.49±0.11 (0.92)	<0.005
**SK-N-SH**	12.09±0.97	**Doxorubicin**	0.45±0.01	0.28±0.002 (0.90)	0.18±0.02 (0.97)	<0.05
		**Cisplatin**	3.84±0.27	1.10±0.17 (0.57)	0.56±0.17 (0.72)	<0.05
		**Etoposide**	17.08±0.93	3.69±0.05 (0.59)	1.68±0.03 (0.84)	<0.005
**SK-N-BE(2)**	10.81±0.62	**Doxorubicin**	2.32±0.56	0.37±0.05 (0.53)	0.16±0.02 (0.81)	<0.005
		**Cisplatin**	23.98±1.3	3.73±0.53 (0.53)	1.42±0.34 (0.80)	<0.005

**Note:** SK-N-SH and SK-N-BE(2) cells were treated with variable concentrations of etoposide, doxorubicin or cisplatin for 48 h in the absence or presence of 4 or 8 µM LY2603618 administered simultaneously. Viable cells were measured by MTT assays and the extent and direction of antitumor interactions between LY2603618 and etoposide, doxorubicin, or cisplatin were determined by using the CompuSyn software. The numbers in the brackets represent for the combination index (CI) values, where CI<1, CI = 1, and CI>1 indicate synergistic, additive, and antagonistic effects, respectively. The data are presented as means±standard errors from at least 3 independent experiments.

When simultaneously combined with etoposide, doxorubicin or cisplatin in the SK-N-BE(2) and SK-N-SH cell lines, LY2603618 potently and synergistically enhanced the effects of the standard chemotherapeutic drugs on cell growth (Table 3). These results are almost identical to that obtained with the combinations of panobinostat and the standard chemotherapeutic drugs. Although LY2603618 significantly enhanced cell death induced by the standard chemotherapeutic drugs in the SK-N-BE(2) cells, its impact on apoptosis induced by the standard chemotherapeutic drugs was minimal (Figures 5C-F). Addition of LY2603618 resulted in decreased phosphorylation of CDC25C(S216) and CDK1(Y15), however, it did not consistently abrogate the G2 cell cycle checkpoint in the SK-N-BE(2) cells, potentially due to down-regulation of total CDK1 (Figures 5E and F).

## Discussion

Neuroblastoma is the most common malignant extracranial solid tumor presenting in childhood. It remains a therapeutic challenge, with long-term survival rates less than 40%. New agents are urgently needed to overcome chemotherapy resistance so as to improve the treatment outcome of this deadly disease. HDACIs have shown great potential for the treatment of cancer [10], [23], [32], [33]. They have been shown to induce cell cycle arrest, differentiation, and apoptosis in cancer cells, but to a much lesser extent in normal cells [10]. Our previous HDACI study in acute myeloid leukemia cells (unpublished data) as well as studies by others in non-small cell lung cancer cells [19] have demonstrated that panobinostat treatments result in down-regulation of CHK1. It has also been demonstrated that CHK1 inhibitors or siRNA treatment sensitize cancer cells, including neuroblastoma, to S/G2 phase-acting agents [8], [9]. This evidence led to our hypothesis that panobinostat may suppress the CHK1 pathway in neuroblastoma cells to enhance the cytotoxicities of etoposide, doxorubicin, or cisplatin.

In this study, we analyzed the molecular effects of combined panobinostat and etoposide, doxorubicin, or cisplatin in high-risk neuroblastoma cell lines. Our MTT assays in neuroblastoma cell lines demonstrated highly synergistic anti-tumor activity when treated with panobinostat and etoposide, doxorubicin, or cisplatin. Trypan blue exclusion, Sub-G1 and AnnexinV flow cytometry analyses, and western blotting revealed enhanced cell death accompanied by cleavage of caspase 3 and PARP when panobinostat was combined with etoposide, doxorubicin, or cisplatin, demonstrating that panobinostat enhanced the standard chemotherapy drug-induced apoptosis. Our additional studies have provided evidence that autophagy probably does not contribute substantially to panobinostat-induced cell death in these cell lines, but cannot be completely ruled out (data not shown). The standard chemotherapy drugs caused activation of the CHK1 pathway, as indicated by increased phosphorylation of CDK1 and CDC25C, as well as increased total levels of CDK1, CHK1 and cyclin B1, which were partially abolished by the addition of panobinostat. Cell cycle analysis revealed that the standard chemotherapeutic drugs mainly caused S and G2/M cell cycle arrest, which was abrogated by the simultaneous addition of panobinostat. Based on the results in Figure 3E, however, it appears that panobinostat is not able to further increase the effect of doxorubicin in cells pretreated with a high doxorubicin dose. It is hypothesized, though not directly investigated here, that higher doses of doxorubicin likely cause activation of the G2/M checkpoint before panobinostat is added. Because the checkpoint is already activated, downregulation of CHK1 by panobinostat may not be sufficient to bypass the cell-cycle arrest. Thus, pretreatment with high doses of doxorubicin followed by treatment with panobinostat is unlikely to result in synergistic anti-tumor activities. Instead, the data suggest that panobinostat can prevent activation of the G2/M checkpoint, and that panobinostat would be better dosed either concurrent with or before standard chemotherapeutic agents in future studies. These results strongly support our hypothesis that panobinostat synergistically enhanced the anti-tumor activity of etoposide, doxorubicin, or cisplatin while down-regulating CHK1 and preventing activation of the G2 cell cycle checkpoint.

To confirm the role CHK1 plays in the synergistic antitumor interactions between panobinostat and the standard chemotherapeutic drugs, we treated neuroblastoma cells with the CHK1-specific inhibitor LY2603618. There was a dose-dependent increase in cell proliferation inhibition, but little to no increase in apoptosis as reflected by minimal increase in cleavage of caspase 3 and PARP. When combined with etoposide, doxorubicin, or cisplatin, synergistic enhancement of growth inhibition was observed by MTT assays and significant enhancement of dead cells by trypan blue exclusion was observed. However, very few cells were detected in Sub-G1 and enhanced cleavage of caspase 3 and PARP were not detected, suggesting that the drug combination did not result in enhanced apoptosis, but rather enhanced growth inhibition and non-apoptotic cell death. SK-N-BE(2) cells treated with LY2603618, alone or in combination with standard chemotherapeutic drugs, displayed increased percentage of cells in S and/or G2/M compared to treatments with vehicle control, etoposide, doxorubicin, or cisplatin alone. The CHK1 inhibitor caused cell cycle arrest but did not enhance etoposide, doxorubicin, or cisplatin-induced apoptosis, suggesting that abrogation of the G2 cell cycle checkpoint may play an important role in the enhancement of etoposide, doxorubicin, or cisplatin-induced apoptosis by panobinostat in the high-risk neuroblastoma cells. Based on our cell cycle data, CDK1 activation occurred in SK-N-BE(2) cells treated with panobinostat and DNA damaging agents. However this was not observed in cells treated with the combination of LY2603618 plus DNA damaging agents, possibly due to decreased levels of total CDK1. While there was some reduction in total CDK1 levels after panobinostat treatment, the cell cycle data suggest that in combinations with panobinostat there remained CDK1 activity whereas the cell cycle arrest seen after treatment with LY2603618 suggests lack of CDK1 activity. Though only speculation, we suspect that while the remaining CDK1 after panobinostat treatment is sufficient to drive cell cycle progression, this is not the case following LY2603618 treatment, possibly due to lower absolute levels. Further investigations are underway to better understand this paradoxical arrest seen after treatment with this CHK1 inhibitor. These results support our hypothesis that panobinostat suppresses the CHK1 pathway and synergizes with standard chemotherapy drugs to induce cell death and/or growth inhibition. However, it does not appear that the enhancement of apoptosis seen in these combinations can be attributed solely to suppression of the CHK1 pathway.

In summary, our study demonstrates that panobinostat suppresses the CHK1 pathway in high-risk neuroblastoma cell lines and synergizes with standard chemotherapy drugs. Our findings need follow up studies in *in vivo* models. Nonetheless, our cell line data suggest a rationale for the combination of panobinostat and standard chemotherapeutic drugs in the treatment of high-risk neuroblastoma.

## Supporting Information

Figure S1 **Representative cell cycle histograms for the SK-N-DZ after panobinostat treatment.** Panels **A**, **B**, **C**, **D**, and **E** show representative cell cycle histograms for SK-N-DZ cells treated with 0 nM, 5 nM, 10 nM, 20 nM 40 nM, respectively. The x-axis shows the PI signal and the y-axis shows the counts for a given PI content. Gates for G1/G0-, S-, G2/M- and subG1- phases were made based on untreated cells and applied to each treatment condition. Quantification of each population is given along the right edge. Histograms were created using FlowJo v7.6.5 (Tree Star; Ashland, OR, USA).
(TIF)
