# Supplementary figures and images for: Panobinostat Synergistically Enhances the Cytotoxic Effects of Cisplatin, Doxorubicin or Etoposide on High-Risk Neuroblastoma Cells
Source: PLoS One. 2013 Sep 30;8(9):e76662. doi: 10.1371/journal.pone.0076662 (PMC3786928; doi:10.1371/journal.pone.0076662)

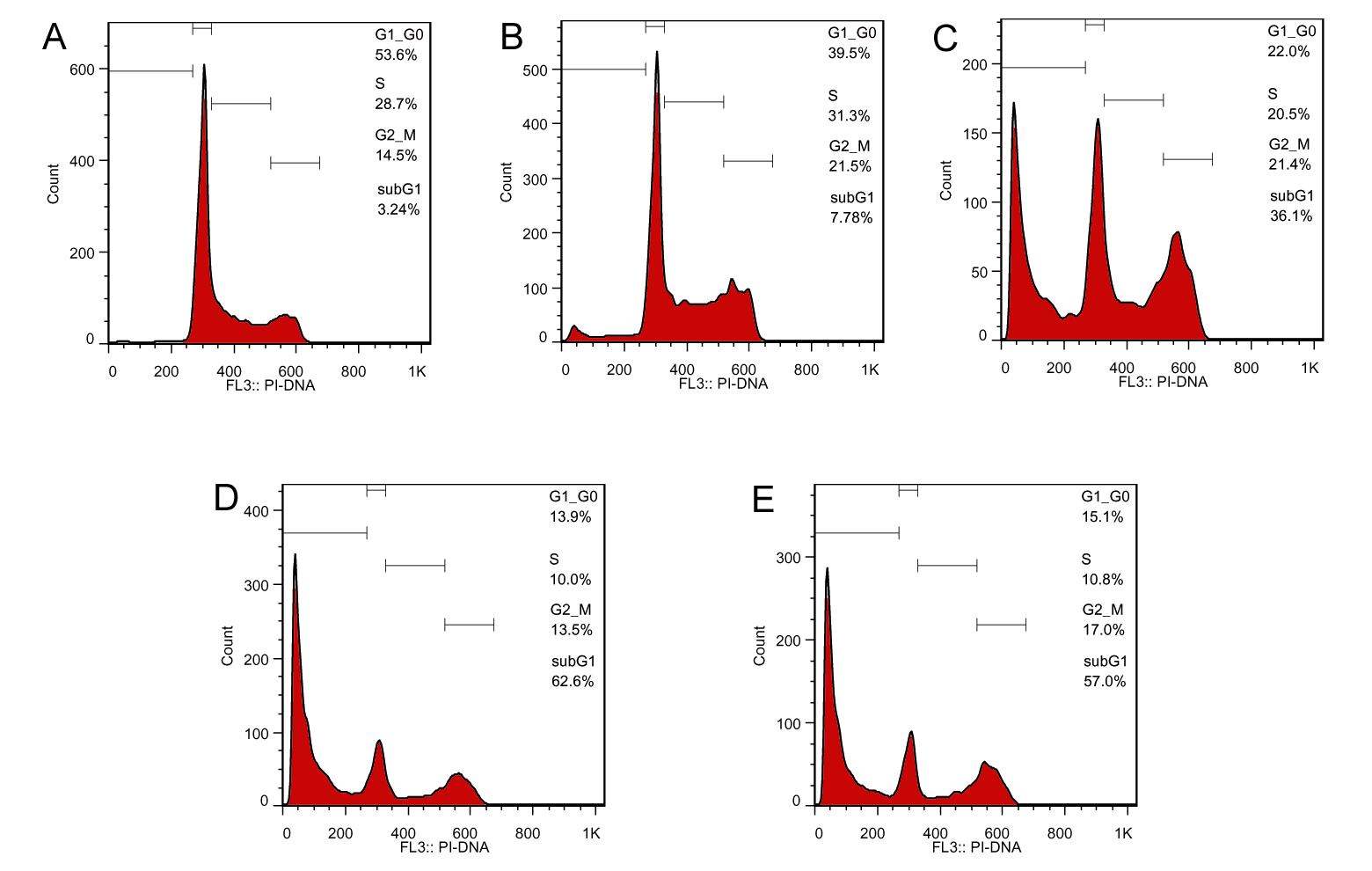

Supplement: Figure S1 — Representative cell cycle histograms for the SK-N-DZ after panobinostat treatment. Panels A, B, C, D, and E show representative cell cycle histograms for SK-N-DZ cells treated with 0 nM, 5 nM, 10 nM, 20 nM 40 nM, respectively. The x-axis shows the PI signal and the y-axis shows the counts for a given PI content. Gates for G1/G0-, S-, G2/M- and subG1- phases were made based on untreated cells and applied to each treatment condition. Quantification of each population is given along the right edge. Histograms were created using FlowJo v7.6.5 (Tree Star; Ashland, OR, USA). (TIF) [file pone.0076662.s001.tif]
